# Evaluation of whole blood CD64 for identifying infection in neonates receiving hospital care

**DOI:** 10.3389/fimmu.2025.1629223

**Published:** 2025-08-18

**Authors:** Naomi E. Spotswood, Peter A. Dargaville, Leah Hickey, Michelle J. L. Scoullar, Riya Palchaudhuri, Shuning Zheng, Timothy Spelman, Suzanne M. Crowe, Hanumesh Kenchapla, James G. Beeson, David A. Anderson

**Affiliations:** ^1^ Burnet Institute, Melbourne, VIC, Australia; ^2^ Faculty of Medicine, Dentistry and Health Sciences, University of Melbourne, Melbourne, VIC, Australia; ^3^ Department of Pediatrics, Royal Hobart Hospital, Hobart, TAS, Australia; ^4^ Menzies Institute for Medical Research, University of Tasmania, Hobart, TAS, Australia; ^5^ Neonatal Medicine, The Royal Children’s Hospital, Melbourne, VIC, Australia; ^6^ Neonatal Research, Murdoch Children’s Research Institute, Melbourne, VIC, Australia; ^7^ Faculty of Medicine, Monash University, Melbourne, VIC, Australia; ^8^ The Walter and Eliza Hall Institute of Medical Research, Melbourne, VIC, Australia

**Keywords:** neonatal sepsis and other neonatal infections, CD64 biomarker, infection diagnostics, bloodstream infection (BSI), whole blood CD64

## Abstract

**Introduction:**

Infection remains one of the most common causes of death in neonates. However, early detection of neonatal infections to inform treatment decisions remains clinically and technically challenging due to the non-specific nature of symptoms, and the lack of a sufficiently accurate diagnostic test. Neonatal infections and sepsis in adults have been associated with increased CD64 expression on neutrophils. We investigated whole blood CD64 (wbCD64) and neutrophil elastase (NE) in neonates who were evaluated and treated for potential infection and evaluated the potential for these biomarkers as diagnostic tools.

**Methods:**

Neonates were prospectively recruited from two neonatal units. Whole blood samples were collected at the time of clinical evaluation for potential infection, if antimicrobials were also initiated. Whole blood CD64 and NE, as a marker of the neutrophil count, were measured by enzyme-linked immunosorbent assays (ELISA). Correlations between wbCD64, NE, and standard hematologic indices were evaluated and diagnostic performance of wbCD64 in relation to infections analyzed using logistic regression and receiver operating characteristic (ROC) curves.

**Results:**

Samples were analyzed from a total of 178 episodes of infection evaluation from 163 neonates. Whole blood CD64 and NE had a positive, non-linear correlation. Infection was diagnosed in 45% (80/178) of episodes, and 31% (55/178) had infection that was microbiologically confirmed. There was no association identified between wbCD64 and infections, and wbCD64 had poor diagnostic performance for infection detection. Evaluation of wbCD64 relative to levels of NE did not improve diagnostic performance. WbCD64 levels were significantly higher among a subgroup of neonates aged >48 hours who had microbiologically-confirmed bacterial bloodstream infections (BSI), with optimal sensitivity and specificity for BSI detection 53% and 87% respectively.

**Conclusion:**

WbCD64 is generally not significantly associated with infection in neonates, but shows some association with bacterial bloodstream infections. The diagnostic performance of wbCD64, with or without NE, does not afford sufficient diagnostic accuracy to aid antimicrobial therapeutic decisions for neonatal infections.

## 1 Introduction

Infections affect millions of neonates each year, and are the third greatest contributor to worldwide neonatal deaths (1–5). Causative pathogens can be bacterial (6), viral (7), fungal (8) or parasitic (9), each with the potential for mortality. Due to immaturity of their innate and adaptive immune defenses, neonates are uniquely susceptible to infections and their associated complications (10–14), particularly if they are born preterm (15). Neonates who require hospital care are additionally exposed to the risk of nosocomial infections (16, 17). The neonate’s host response to infection is as yet incompletely understood (6). Available evidence delineates the release of both pro-inflammatory and anti-inflammatory mediators, with dysregulation in this response associated with multi-organ dysfunction that can lead rapidly to death (6, 18, 19). Variations in neonatal host responses to infection are observed with both preterm birth and postnatal age. Premature neonates have heightened infection susceptibility compared to term neonates, and differences in their immune response (15, 19). Immune response differences are also observed between neonates with infections in their first few days, referred to as early-onset infections, compared to late-onset infections (20). Regardless of age or gestation at birth, neutrophils play an early and critical role in the neonate’s infection response (10, 21). When activated by the presence of infection, they express CD64, also referred to as Fc gamma receptor 1 (FCγR1) (22).

Early and accurate identification of infections in neonates is a critical first step in clinical care, affording the best opportunity for survival and recovery through prompt antimicrobial and supportive treatments (23). However, this remains a major challenge even for experienced clinicians as features of infections in neonates are subtle, and can mimic other non-infectious diagnoses (24). Available diagnostic tests that assist this process include microbiologic culture and polymerase chain reaction (PCR) for pathogen identification, hematologic indices and biomarkers associated with infection or inflammation such as C-reactive protein (CRP) and procalcitonin (25). These are largely laboratory-based, and can take hours to days from the time of specimen collection to a result. Therefore, a decision to start empiric treatment is usually made before all test results are available (26, 27). Consequently, many neonates who receive antimicrobial treatment are ultimately found not to have infection (28, 29), and have thus been unnecessarily exposed to antimicrobials. Antimicrobial treatment in the absence of infection carries risks of iatrogenic harm, including medication errors, delayed breastfeeding, and increased mortality for preterm neonates (30–32). Hence an accurate point of care test for neonatal infections is needed, to better achieve early treatment for true infections and avoid unnecessary antimicrobial exposure for cases where infection is not the diagnosis. Such a test would avoid the need for specialized laboratory equipment and could be particularly valuable in remote and resource-constrained settings (26, 33, 34).

Many biomarkers related to the neonate’s host response to infection have been suggested as potential point of care tests (26) including C-reactive protein, presepsin, procalcitonin, interleukins 6 and 8, tumor necrosis factor-alpha, and neutrophil CD64 (26, 33). While some show promise, no single test or test combination has yet demonstrated the diagnostic accuracy required for widespread point of care diagnostic use. Neutrophils typically express CD64 at low levels in a resting state, and rapidly upregulate expression in the presence of infection or inflammation, although data from diverse populations are limited (35, 36). Raised neutrophil CD64 measured using flow cytometry has been reported in neonates receiving hospital care who have infections, including both invasive bacterial infections and infections diagnosed without microbiologic confirmation due to clinical signs and/or other biomarkers of inflammation (36–41). However, flow cytometry requires sophisticated laboratory equipment, trained technicians, and is generally performed in daytime working hours, therefore limiting its feasibility for the rapid diagnosis of infection (22). Monocytes and macrophages express surface CD64 constitutively, although expression level varies between subsets (35, 42), with some upregulation observed during neonatal infections (43).

Whole blood testing of CD64 could allow for a simplified point of care test approach, avoiding the need for specialized laboratory equipment. However, greater knowledge on whole blood CD64 (wbCD64) levels in neonates is needed. As neonates have a propensity to neutropenia (44), the influence of total neutrophil count on CD64 levels requires evaluation, as lower levels in whole blood could occur where the neutrophil count is low despite neutrophil CD64 upregulation (45, 46). Further, wbCD64’s potential diagnostic performance needs evaluation across the broad range of clinical situations where a neonatal infection point of care test might be used. Such situations encompass term and preterm neonates, early-onset and late-onset infections, and infections that are acquired in communities and in hospitals (47). In adults, wbCD64, measured by enzyme-linked immunosorbent assay (ELISA) of lysed whole blood was evaluated in patients with sepsis versus controls, combined with a surrogate marker of neutrophil count, neutrophil elastase (NE) (45, 46, 48). Elevated levels of wbCD64 relative to NE were found in adults with sepsis (45). Using cut-offs derived from a non-linear relationship between wbCD64 and NE, a pilot study in adults identified sepsis with 100% sensitivity and 94% specificity relative to controls (46). This approach demonstrated the potential for the combined measurement of wbCD64 and NE as a point of care test. Whether this approach can be used in neonates has not previously been reported. Therefore, in this exploratory study we analyzed wbCD64 and NE in neonates with and without infection and provide the first report of wbCD64 measurement in this population. We aimed to (1) determine the ranges of wbCD64 and NE in neonates who are evaluated and treated for possible infection, (2) understand the relationship between wbCD64 and NE in neonates, and (3) evaluate the diagnostic potential of wbCD64 in isolation and combined with NE for the early detection of neonatal infection.

## 2 Materials and methods

### 2.1 Study setting and approvals

This prospective observational cohort study recruited participants between December 2018 and February 2021 from two tertiary Australian neonatal units: The Royal Children’s Hospital (RCH) and Royal Hobart Hospital (RHH). Some interruptions to recruitment occurred during the early stages of the SARS-Cov-2 pandemic. Both units admit neonates from the emergency department when needed, RCH is a referral center for neonates who need advanced ventilation techniques or surgery and RHH is a perinatal and surgical center, predominantly caring for preterm neonates. Ethical approval was provided by each center’s Human Research Ethics Committees (HREC) (RCH HREC: 38207; University of Tasmania HREC: H0018176).

### 2.2 Eligibility criteria and recruitment

Neonates were included if informed written consent from their guardian was provided and they were: i) aged <28 days or 44 weeks corrected gestation age; ii) evaluated by the hospital’s clinical staff for potential infection and commenced antibiotics; and iii) a blood specimen as part of clinical care was collected in an ethylenediaminetetraacetic acid (EDTA) tube for a full blood count in the period between four hours prior to and two hours after antibiotic commencement. Exclusion criteria included a known diagnosis of a congenital neutropenia syndrome, receiving extra-corporeal life support, and prior administration of parenteral antibiotics for more than 2 hours for the treatment of an infection at the time of evaluation when the EDTA tube was collected. For neonates with multiple evaluations across their hospital admission, further samples (if available) along with related data were collected. Recruitment aimed to reach equal numbers of evaluation and treatment episodes for term and preterm neonates. Study participation did not impact clinical care and did not involve any blood collection additional to routinely collected tests.

### 2.3 Specimen processing and analysis

The whole blood samples, which in this study had in the first instance been collected for clinical purposes, were initially stored at 4°C in local laboratories for 7 days per national laboratory accreditation requirements, and within the week thereafter frozen at -80°C prior to batch analysis. CD64 and NE were each measured using single batches of commercially available enzyme-linked immunosorbent assay (ELISA) kits (Cloud Clone Corp. and R&D Systems, respectively), with detailed methods available in **Supplementary 1**. Investigators performing these assays were unaware of infection status.

### 2.4 Data collection, definitions, subgroups and outcome groups

The Strengthening the Reporting of Observational Studies in Epidemiology for Newborn Infection (STROBE-NI) statement informed preparation of this study (49). Data were collected using REDCap (Research Electronic Data Capture) (50, 51) from medical, laboratory and radiology records at each site. The first day of life was designated day 0 (49). Prematurity was defined as birth at or prior to 37 weeks’ gestation, and the timepoint differentiating early and late-onset neonatal infections was set at 48 h per the definition of the Australian and New Zealand Neonatal Network (ANZNN) (52). Birthweight percentiles were calculated per the Fenton 2013 growth charts (53). Late-onset infections were categorized as healthcare associated if the neonate had been admitted to hospital for 48 hours or greater at the time of their evaluation, and community-acquired otherwise (47). All clinical laboratory results (hematologic indices, immature to total neutrophil ratio, C-reactive protein and microbiologic identification tests) were sourced from the medical record. The neutrophil to monocyte ratio was calculated as (neutrophil count x10^9^/L)/(monocyte count x10^9^/L).

Two clinician investigators performed the outcome categorization for each episode. Information available to these clinician investigators included the each episode’s classification per the ANZNN definitions for neonatal bloodstream infection, meningitis, viral infection and necrotising enterocolitis (52), a published set of consensus criteria for neonatal infection which includes criteria for attribution of a diagnosis of infection in the absence of microbiologic confirmation (54), and review of medical, radiological and laboratory records. The presence of alternative diagnoses to infection was also considered and recorded for each episode. Where there was disagreement between the reviewers as to the outcome category, the opinion of a third clinician investigator was sought. Each evaluation and treatment episode was assigned to one of the following pre-specified hierarchical outcome categories:

1. Culture confirmed bloodstream infection, bacterial.
2. Culture confirmed bloodstream infection, fungal.
3. Culture confirmed meningitis, bacterial.
4. Culture confirmed meningitis, fungal.
5. Microbiologically confirmed meningitis or encephalitis, viral.
6. Microbiologically confirmed respiratory tract infection, bacterial.
7. Microbiologically confirmed respiratory tract infection, fungal.
8. Microbiologically confirmed respiratory tract infection, viral.
9. Other microbiologically confirmed infection.
10. Culture negative bloodstream infection.
11. Culture negative meningitis.
12. Culture negative respiratory tract infection.
13. Necrotising enterocolitis.
14. Other infection without microbiologic confirmation nonetheless suspected to be a culture negative infection.
15. No infection.

Details regarding each evaluation and treatment episode’s pathogen(s) and infection site(s) were recorded. Using the above outcome categories, the following infection outcome groups were formed for analysis:

- Any infection: any of outcome categories 1 to 14.
- Microbiologically confirmed infection: any of outcome categories 1 to 9.
- Bacterial infection: any of outcome categories 1, 3, 6, or 9 (if a bacterial pathogen was specified).
- Viral infection: any of outcome categories 5, 8, or 9 (if a viral pathogen was specified).
- Bacterial bloodstream infection: outcome category 1.
- No infection: outcome category 15.

### 2.5 Statistical analysis

Statistical analysis was performed in in Stata (StataCorp. 2023. Stata Statistical Software: Release 18. College Station, TX 77845, USA; StataCorp LLC). Missing data are indicated directly in the results, without imputation in the analyses. Normality of continuous variables was tested using the Shapiro-Wilk test.

Subgroup analyses were performed by gestation at birth and the age at the time of infection evaluation:

- Term (>37 weeks’ gestation at birth) and preterm (≤37 weeks’ gestation at birth).
- Early-onset (<48 hours of age at the time of evaluation) and late-onset (≥48 hours of age at the time of evaluation).

These subgroup categories were not mutually exclusive.

Spearman’s rank correlation was used to evaluate relationships between monotonic, non-parametric continuous variables, specifically wbCD64 with NE, neutrophil counts and monocyte counts. Univariate logistic regression was used to compare continuous and categorical variables including wbCD64, NE hematologic indices and CRP between subgroups.

Infection outcome group analyses of wbCD64, hematologic indices and CRP were performed using univariate and multivariate logistic regression. Covariates with a p-value of <0.1 in univariate regression analyses were included in the multivariate logistic regression models. For all logistic regression analyses, adjustment for clustering was undertaken to account for neonates with multiple evaluation episodes where appropriate. Receiver operating curves (ROC) were generated for each outcome group, with Youden’s index used to identify optimal diagnostic cut points with associated sensitivity and specificity (55).

Each infection outcome group evaluation comprised analysis of the specified outcome group against all other evaluations (those with no infection and those with other infection group outcomes combined). Evaluation of microbiologically-confirmed infections was limited to the subgroup of late-onset evaluations, as none occurred in evaluations at <48 hours of age. Ethnicity was not well-documented in medical records, therefore analysis by ethnicity was not possible. Two episodes of evaluation for infection were excluded from this analysis due the sample having insufficient volume and 20 where it was noted that the neonate had received antimicrobials outside of the protocol’s timeframe.

## 3 Results

### 3.1 Study population and infection outcomes

Across the two study sites during periods of active study recruitment, 367 episodes of evaluation for infection and antimicrobial treatment were identified, with 283 deemed potentially eligible for inclusion in the study. Of these, 178 eligible samples with a sufficient volume for analysis from 163 neonates were included in this analysis. Eleven neonates (6.7%) had more than one episode of infection evaluation and treatment with an associated blood sample and clinical data included (range 2 to 3).

Demographic characteristics are detailed in **Table 1**, and infection outcome groups in **Table 2**. Across all episodes, the median gestation was 36 weeks (IQR 30 to 39) and median age was 9 days (IQR 0 to 24). Infection was identified in 80 (45%) of the 178 evaluations. Of these, 55 (69%) were microbiologically confirmed, all in the subgroup of evaluations for possible late onset neonatal infections (age ≥48 hours). Of microbiologically confirmed infections, 27 were bacterial (49%) and 28 were viral (51%). Fifteen of the bacterial infections were bloodstream infections (56%). Of infections from late-onset evaluations (≥48 hours of age), 46 (61%) were community-acquired and 30 (39%) were hospital-acquired. Identified pathogens for microbiologically confirmed infections are listed in **Supplementary 2**.

**Table 1:** Neonatal population characteristics.

Characteristic	All evaluations	Any infection	No infection
Preterm (≤37 weeks)*** n/N (%)	107/178 (60.1)	42/80 (52.5)	65/98 (66.3)
Birthweight in grams median (IQR)*	2635 (1340-3290)	2820 (1340-3465)	2485 (1340-3240)
Small for gestational age* n/N (%)	23/175 (13.1)	10/77 (13.0)	13/98 (13.3)
Female sex n/N (%)	71/178 (39.9)	32/80 (40.0)	39/98 (39.8)
Age ≥48 hours n/N (%)**, ***	109/175 (62.3)	76/79 (96.2)	33/96 (34.4)
Hospital admission >48 hours prior to evaluation*** n/N (%)	55/109 (50.5)	30/76 (39.5)	25/33 (75.8)

*Classified per Fenton’s 2013 Growth Charts (53). Three neonates did not have birthweight data.

**Three neonates had insufficient data to categorize age in hours.

***Characteristics with a p-value of <0.1 and included in multivariate logistic regression analyses.

**Table 2:** Infection outcomes.

Infection outcome	All evaluations	Gestation at birth subgroups*		Age at the time of evaluation subgroups*			
		Preterm	Term	Early-onset (<48 hours) *	Late-onset (≥48 hours) **		
					All	Community acquired***	Hospital acquired****
No infection n/N (%)	98/178 (55.1)	65/107 (60.8)	33/71 (46.5)	63/66 (95.5)	33/109 (30.3)	8/54 (14.8)	25/55 (45.5)
Any infection n/N (%)	80/178 (44.9)	42/107 (39.3)	38/71 (53.5)	3/66 (4.6)	76/109 (69.7)	46/54 (85.2)	30/55 (54.6)
Microbiologically-confirmed infections n/N (%)	55/178 (30.9)	25/107 (23.4)	30/71 (42.3)	0/66 (0)	55/109 (50.5)	36/54 (66.7)	19/55 (34.6)
Bacterial infections n/N (%)	27/178 (15.2)	19/107 (17.8)	8/71 (11.3)	0/66 (0)	27/109 (24.8)	12/54 (22.2)	15/55 (27.3)
Viral infections n/N (%)	28/178 (15.7)	6/107 (5.6)	22/71 (31.0)	0/66 (0)	28/109 (25.7)	24/54 (44.4)	4/55 (7.3)
Bacterial bloodstream infections n/N (%)	15/178 (8.4)	13/107 (12.2)	2/71 (2.8)	0/66 (0)	15/109 (13.8)	4/54 (7.4)	11/55 (20.0)

*These subgroup categories were not mutually exclusive.

**Three had insufficient data to categorize age in hours, including one case of infection not microbiologically confirmed.

***Not hospital admitted for >48 hours prior to evaluation.

****Hospital admission >48 hours prior to evaluation.

Fewer infections occurred for evaluations in neonates who were preterm than term (p = 0.061). More infections occurred for late-onset than early-onset evaluations (p <0.001) (**Table 1**). For late-onset infection evaluations, neonates admitted to hospital for >48 hours had fewer infections (p = 0.001) (**Table 2**).

### 3.2 Whole blood CD64 and neutrophil elastase

Median whole blood CD64 was 139.1ng/mL (IQR 102.8ng/mL to 172.7ng/mL). Preterm neonates had higher wbCD64 values than term neonates (p=0.001). Early-onset evaluations (<48 hours of age) had higher wbCD64 values than late onset-evaluations (≥48 hours of age) (p = 0.004) (**Table 3**).

**Table 3:** Whole blood CD64 levels and associations with infection outcomes.

Evaluation group or subgroup	Infection outcome N=evaluations	wbCD64 (ng/mL) median (IQR)	Unadjusted*		Adjusted*	
			OR (95% CI) per 10ng/mL increment	P-value	OR (95% CI) per 10ng/mL increment	P-value
All 178 evaluations for 163 neonates**	All evaluations N=178	139.1 (102.8-172.7)	n/a			
	No infection N=98	142.2 (107.5-172.1)				
	Any infection N=80	132.0 (95.8-175.8)	0.98 (0.93-1.03)	0.396	1.06 (0.98-1.14)	0.146
Preterm 107 evaluations for 92 neonates**	All evaluations N=107	140.7 (107.5-214.0)	n/a			
	No infection N=65	141.5 (112.4-209.7)				
	Any infection N=42	140.7 (94.6-231.7)	1.00 (0.95-1.05)	1.00	1.04 (0.96-1.12)	0.34
Term 71 evaluations for 71 neonates**	All evaluations N=71	128.3 (97.5-157.9)	n/a			
	No infection N=33	145.8 (101.2-159.7)				
	Any infection N=38	120.0 (96.9-145.4)	0.94 (0.84-1.06)	0.331	1.18 (0.97-1.44)	0.101
Early-onset 66 evaluations for 66 neonates	All evaluations N=66	153.0 (124.9-203.2)	n/a			
	No infection N=63	151.3 (123.1-178.7)				
	Any infection N=3	254.4***	1.27 (1.03-1.56)	0.024	1.61 (0.98-2.64)	0.059
Late-onset 109 evaluations for 97 neonates**	All evaluations N=109	129.8 (94.0-158.8)	n/a			
	No infection N=33	134.0 (88.1-145.8)				
	Any infection N=76	127.3 (94.5-163.5)	1.01 (0.95-1.08)	0.757	1.06 (0.98-1.15)	0.169
	Microbiologically confirmed infections N=55	126.3 (94.6-166.0)	1.01 (0.95-1.08)	0.715	1.07 (0.98-1.16)	0.145
	Bacterial infections N=27	145.4 (110.8-231.7)	1.09 (1.01-1.18)	0.020	1.09 (0.99-1.20)	0.066
	Viral infections N=28	115.5 (88.9-139.1)	0.91 (0.85-0.98)	0.009	0.96 (0.88-1.04)	0.314
	Bacterial bloodstream infections N=15	181.0 (112.0-232.6)	1.11 (1.03-1.20)	0.008	1.08 (1.00-1.16)	0.043

*Logistic regression analyses with the presence of the infection outcome the dependent variable, compared to all other evaluations not resulting in that outcome, clustered where appropriate to account for neonates who contribute data for separate sepsis evaluations. Adjusted (multivariate) analyses included: preterm birth, age ≥48 hours for the entire cohort, preterm and term subgroups; preterm birth for the <48 hour evaluations subgroup; both preterm birth and hospital admission >48 hours prior to evaluation for the >48 hour evaluations subgroup. **Three had insufficient data to categorize age in hours, thus are not included in the age at time of evaluation subgroups, and all adjusted analyses. ***IQR not reported as n=3.

Median NE was 5.1 µg/mL (IQR 2.3 µg/mL to 10.2 µg/mL). NE for preterm neonates did not significantly differ from term neonates (median 3.8 µg/mL [IQR 1.7 to 8.9 µg/mL] versus median 6.2 µg/mL [IQR 3.2 to 11.3 µg/mL] respectively; p=0.411). Early-onset evaluations had similar NE values to late-onset evaluations (median 5.6 µg/mL [IQR 2.2-12.2 µg/mL] versus median 4.6 µg/mL [IQR 2.3-8.5 µg/mL] respectively; p=0.583).

### 3.3 Hematologic indices and CRP

No statistically significant differences were observed in total white cell, neutrophil and monocyte counts quantified as part of the full blood examination between neonates with and without infection (**Supplementary 3**). The median ratio of neutrophils to monocytes was 4.2 (IQR 2.9 to 6.6), with no statistically significant difference in this ratio between neonates with and without infection (median 3.8 [IQR 2.4 to 6.5] versus median 4.6 [IQR 3.0 to 6.9] respectively; p=0.744). The immature to total ratio (ITR) of neutrophils, often reported as a marker of infection or inflammation as part of the full blood examination, was measured in 113 episodes. ITR was higher in neonates with infections than without (median 0.13 IQR 0.05-0.33 versus 0.10 IQR 0.04-0.19, p=0.001). CRP, also often reported as a marker of infection or inflammation, was measured in 142 episodes. CRP was greater than 20 mg/L in 20/60 episodes with infection (33%) versus 7/82 episodes without infection (9%) (p=0.151), with sensitivity and specificity of 33.3% and 91.5%, respectively (**Supplementary 3**).

### 3.4 Whole blood CD64: relationships with neutrophil elastase, neutrophil and monocyte counts

**Figure 1:**
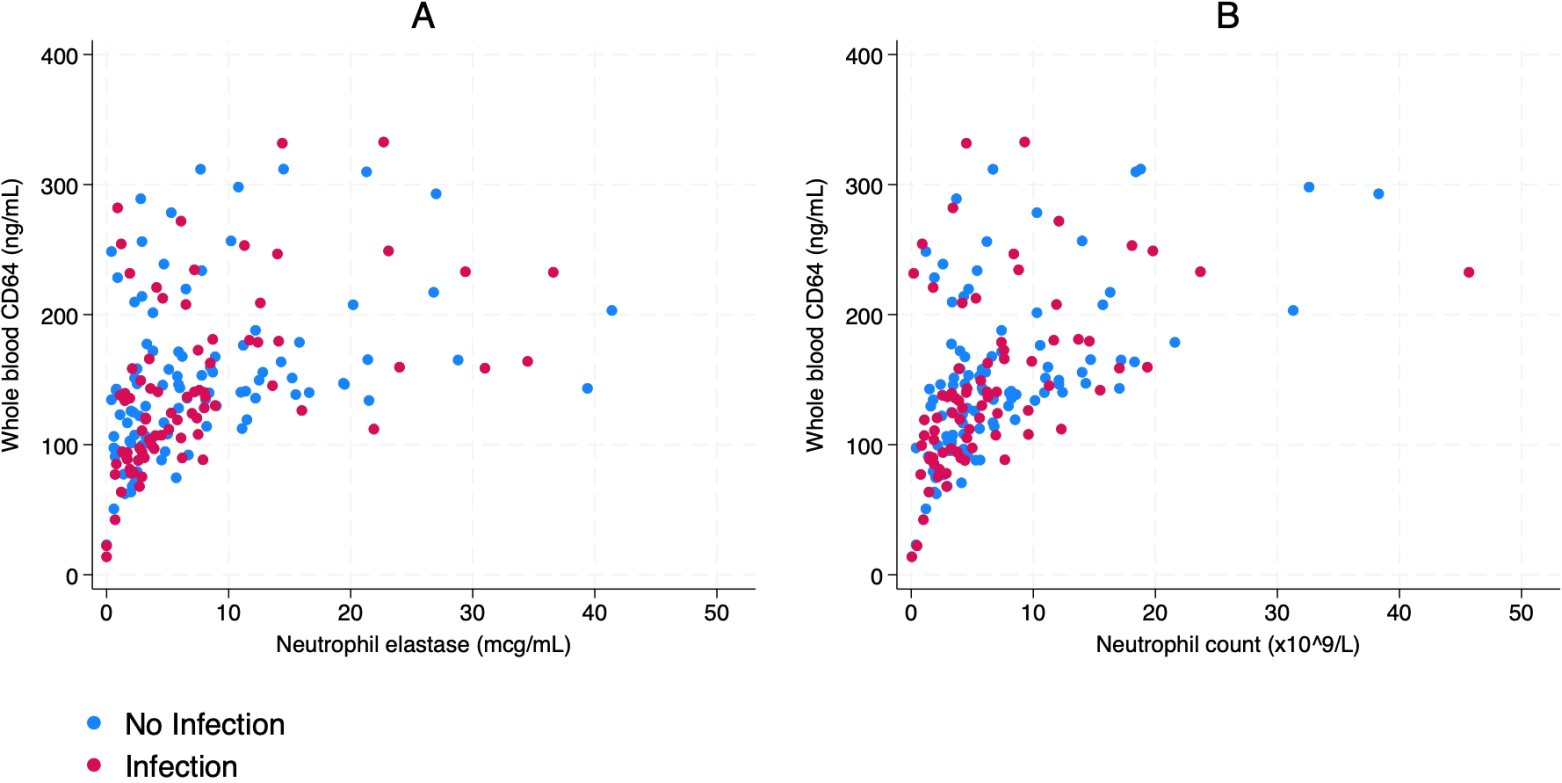
**(A)** Whole blood CD64’s correlation with neutrophil elastase; **(B)** Whole blood CD64’s correlation with neutrophil count.

There was a moderate positive, non-linear correlation between wbCD64 and NE (r_s_=0.52, p<0.001), displaying the same overall pattern as that observed in a prior study of adults (45, 46, 48). This pattern was observed for neonates with and without infection in the whole cohort (r_s_=0.57, p<0.001 and r_s_=0.48, p<0.001, respectively) (**Figure 1**) and in each of the subgroups (term and preterm neonates; early-onset and late-onset evaluations) (**Supplementary 4**).

Whole blood CD64 and neutrophil count displayed a moderate, positive, non-linear correlation, with a similar pattern to that observed between NE and wbCD64 (r_s_=0.57, p<0.001). This was observed for neonates with and without infection (r_s_=0.59; p<0.001 and r_s_=0.54; p<0.001, respectively) (**Figure 1**). Monocyte count and wbCD64 displayed a weak, positive, non-linear correlation (r_s_=0.37; p<0.001), which was observed for neonates with and without infection (r_s_=0.37; p<0.001 and r_s_=0.34; p<0.001, respectively; **Supplementary 5**).

No pattern of diagnostic discrimination between cases with and without any infection was observed based on wbCD64’s non-linear relationship with NE for all evaluations or for the subgroups (term and preterm neonates; early-onset and late-onset evaluations) (**Figure 1**, **Supplementary 4**).

### 3.5 Associations between whole blood CD64 and infections

The presence of any infection, inclusive of all infection types, was analyzed against no infection for all 178 evaluations. Whole blood CD64 was not significantly associated with the presence of any infection (**Table 3**). Similarly, no association between wbCD64 and the presence of any infection was observed in any of the four subgroups in adjusted analyses (term and preterm neonates; early-onset and late-onset evaluations) (**Table 3**).

Microbiologically-confirmed infections were analyzed for the late-onset evaluations group, with each group analyzed against all other outcomes (no infection or other infection types combined). Higher wbCD64 was associated with microbiologically confirmed bacterial bloodstream infections (BSI), after adjustment for preterm birth and hospital admission for greater than 48 hours prior to evaluation (**Table 3**). Other microbiologically confirmed infection outcomes did not show significant associations with wbCD64 in adjusted analyses (**Table 3**).

### 3.6 Diagnostic performance of whole blood CD64 for infections

**Figure 2:**
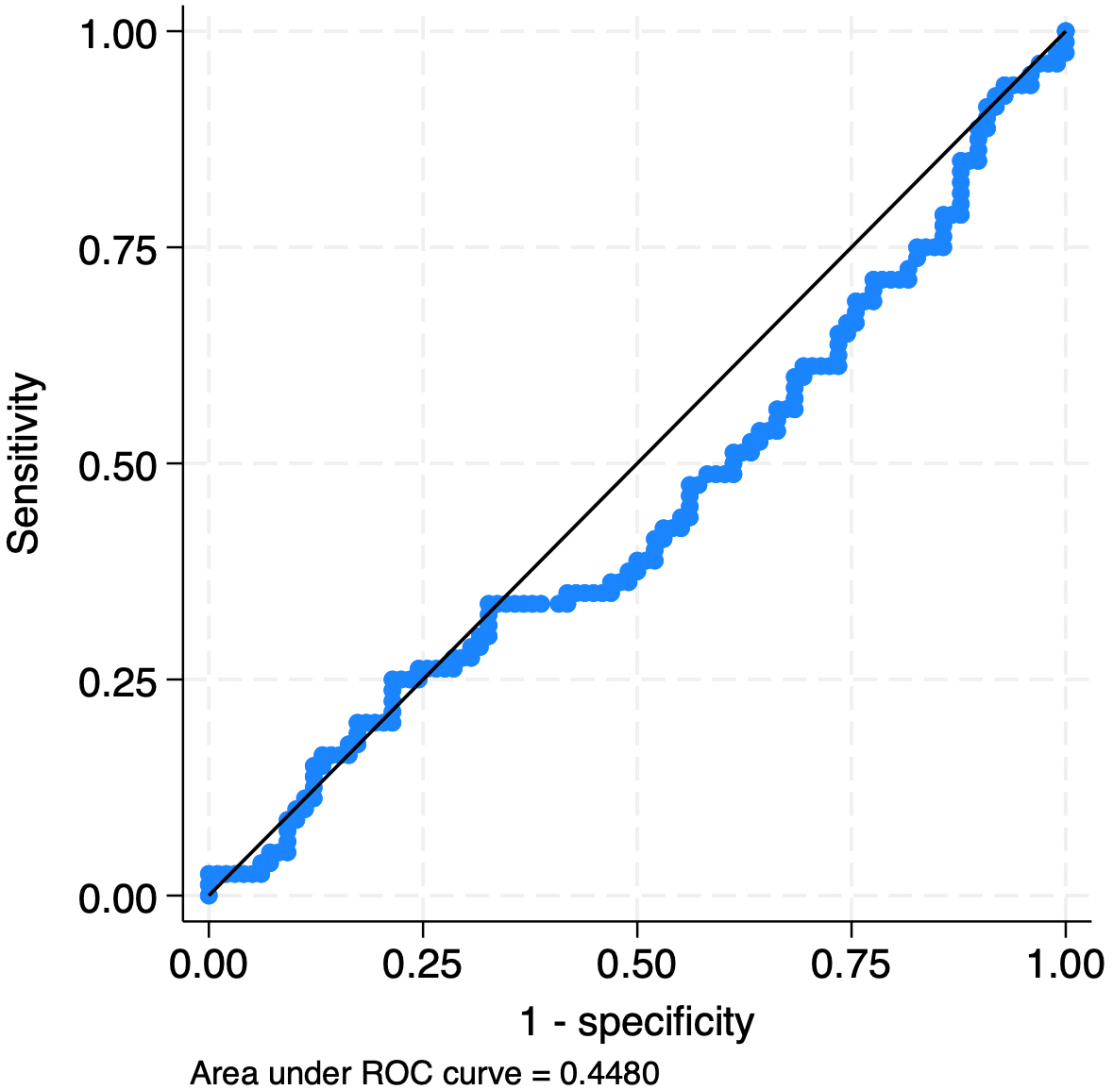
Receiver operating curve for whole blood CD64’s detection of any infection.

**Figure 3:**
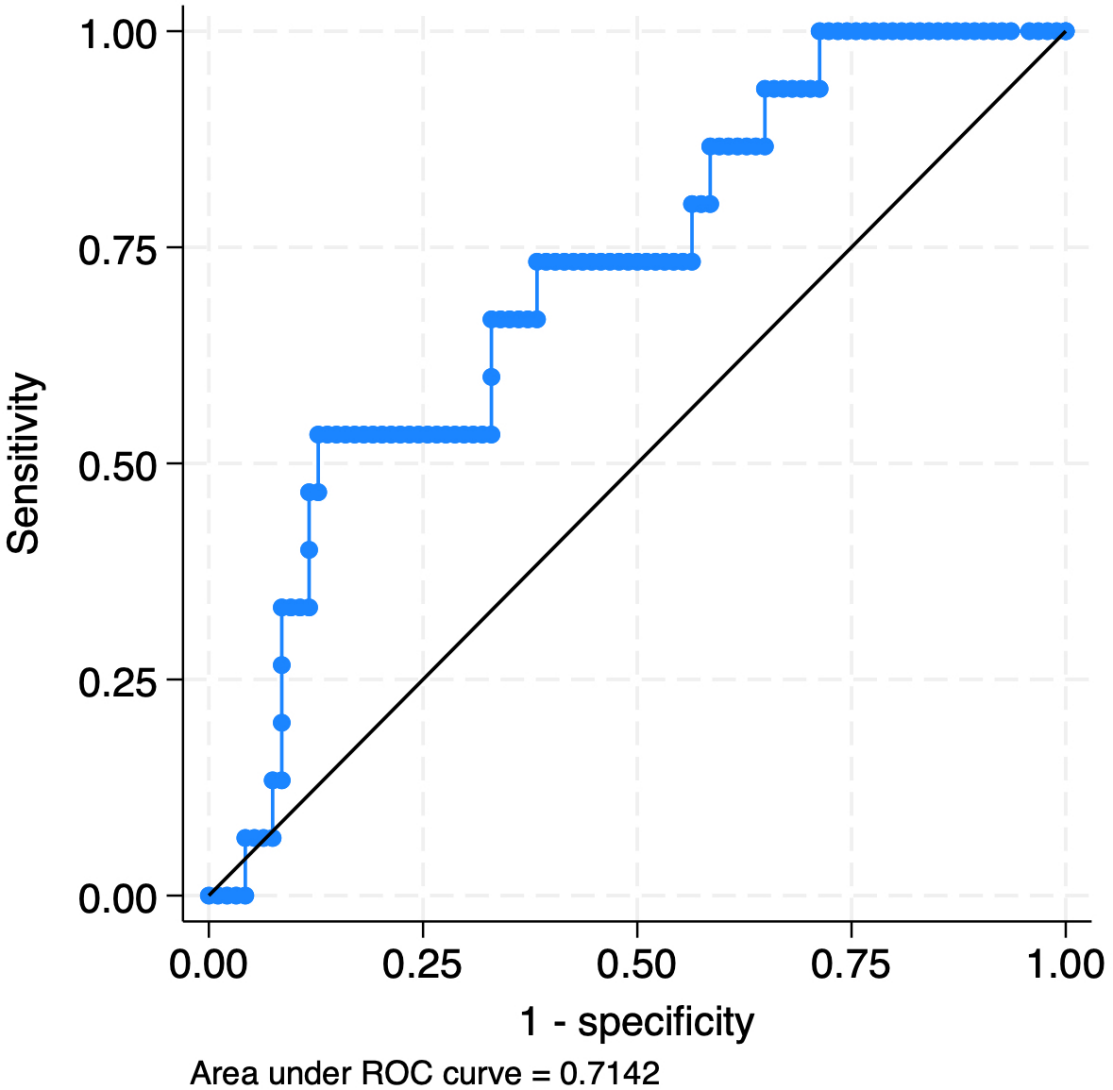
Receiver operating curve for whole blood CD64’s detection of culture-confirmed bacterial bloodstream infections in late-onset evaluations.

Whole blood CD64 showed poor diagnostic performance for the detection of any infection compared to no infection for the entire cohort of 178 evaluations, with a ROC area under the curve (AUC) of 0.45 (**Figure 2**; **Table 4**). For early-onset evaluations (<48 hours of age), a ROC curve was not generated and sensitivity and specificity were not calculated due to the low number of infections (n=3). The AUC for bacterial bloodstream infections for late-onset evaluation (≥48 hours of age) was 0.71 (**Figure 3**, **Table 4**). A cut-off point of 180.3 ng/mL provided 53% sensitivity and 87% specificity for the detection of bacterial BSIs compared to evaluations with any other infection outcome or no infection. Given no other AUCs reached an acceptable value of ≥0.7 (56), optimal sensitivity and specificity cut-offs were not calculated.

**Table 4:** Receiver operating curve data for wbCD64 and infection outcomes.

Evaluation group or subgroup	Infection outcome	AUC (95% CI)
All evaluations	Any infection	0.45 (0.36-0.53)
Preterm	Any infection	0.49 (0.37-0.61)
Term	Any infection	0.41 (0.27-0.55)
Late-onset	Any infection	0.53 (0.41-0.66)
	Microbiologically confirmed infections	0.54 (0.43-0.65)
	Bacterial infections	0.67 (0.56-0.79)
	Viral infections	0.38 (0.27-0.49)
	Bacterial bloodstream infections	0.71 (0.58-0.85)

AUC, Area under the curve; CI, Confidence interval.

## 4 Discussion

In this study we provide to our knowledge the first analysis of wbCD64 levels in neonates evaluated for infection, relative to neutrophil and monocyte levels, and an assessment of the potential diagnostic value of this marker for the early identification of infection. We report, for the first time, a significant non-linear correlation between wbCD64 and NE in neonates, similar to that observed in adults (46, 48). Additionally, we confirm that the relationship between wbCD64 and NE (used as a biomarker surrogate of neutrophil count) closely approximates the relationship between wbCD64 and neutrophil count, a finding not evaluated in previous work in an adult population. We found that wbCD64 and NE do not provide sufficient diagnostic accuracy to identify infection at the time of clinical evaluation in a heterogeneous population of neonates receiving hospital care.

Our study’s key finding is that wbCD64 either in isolation or relative to NE provides no clear diagnostic discrimination between neonates who do and do not have infections. This contrasts with prior work with wbCD64 in adults (46, 48), and neutrophil CD64 expression by flow cytometry in neonates (37, 38). We postulate that potential reasons for this finding include our study population’s intentional exclusion of neonates without any clinical concerns for infection, the timing of sample collection, sepsis definition differences between neonates and adults, and the relative contribution of basal monocyte CD64 expression in neonates.

For this study, we evaluated the performance of wbCD64 and NE in a patient population for whom a point of care test might feasibly be employed. For a well or medically stable neonate, the question of whether infection is present does not need to be answered by a diagnostic test; therefore we did not include healthy neonatal controls in our study. In a prior exploratory study of CD64 in adult patients, controls were healthy adults and patients admitted to an intensive care unit without sepsis. Similarly, in some previous neonatal neutrophil CD64 diagnostic studies, the control group has been comprised of neonates without any clinical concern for infection (40, 57–61). It is possible that this approach magnifies the diagnostic potential of this biomarker for sepsis detection, as neonates who are medically complex or hospitalized may be more likely reasons other than infection for increased CD64 expression than healthy controls. In a surveillance study employing daily neutrophil CD64 measurements in very low birthweight infants, unexplained CD64 activation was observed to occur in some infants without infection (41). In another surveillance study of CD64 measurements for late onset infections, maternal inflammation, intraventricular hemorrhage and mechanical ventilation initiation were each postulated as potential causes for elevated CD64 expression observed in the absence of infection (62). In our study, it is possible that other processes inherent in non-infective conditions that prompted clinician suspicion for infection may have had an impact on the expression of neutrophil CD64 and wbCD64 levels. However, there are also some studies which, similar to our design, evaluated neutrophil CD64 solely in neonates who had commenced antimicrobial therapy, and observed higher neutrophil CD64 values in neonates with infection (43). Thus, we cannot assume that our study design is the sole reason for wbCD64’s poor diagnostic discrimination in our cohort.

Infections in neonates can be life-threatening without prompt antimicrobial treatment. For a point of care test to effectively support a decision to start treatment, its accuracy must be optimal at the first point of clinical evaluation. In the prior work evaluating wbCD64 in an adult population, specimens were collected within 48 hours of antimicrobial commencement (46). By contrast, in this study, we limited our analysis to samples collected within a short window of time near the start of antimicrobial therapy, emulating as best we could the process of a potential point of care test being performed at the time when the clinician needs to decide if they should start antimicrobial treatment. Neutrophil CD64 is often presented as a potential point of care test biomarker due to its higher likelihood of being elevated early in an infectious illness than other more routinely used biomarkers including C-reactive protein (59, 61). However, in some reports of neutrophil CD64’s diagnostic accuracy in neonates, sample timing in relation to symptom onset or antimicrobial therapy commencement is not clearly specified, or could occur in a longer timeframe than specified in our study, up to 24 hours following symptom onset (41, 60). Further, peak neutrophil CD64 levels may occur up to 24 hours after the onset of symptoms (57) rather than at the outset of an infectious illness as has been observed in children and adults (43). From our analysis, with a strict time period during which included samples were collected, wbCD64 does not appear to be a biomarker likely to aid early treatment decisions. Changes in wbCD64 over the course of a neonatal infection may merit further study, as diagnostic discrimination later in the course of illness may aid other treatment decisions, such as stopping antimicrobials.

In this analysis, we intentionally use the term infection rather than neonatal sepsis because there is a current lack of a robust and universal definition for neonatal sepsis. Defining sepsis in neonates is a long-standing challenge (63, 64), and varied sepsis or infection definitions are used across neonatal neutrophil CD64 literature (41, 43, 57, 58, 60). Sepsis in adults is defined as “…life-threatening organ dysfunction caused by a dysregulated host response to infection” (65). While a neonatal sepsis definition that includes organ dysfunction for preterm infants has been developed (66, 67), this definition is not yet widely used for clinician-led sepsis identification across the world. Infections in neonates generally require antimicrobial therapy regardless of whether there is organ dysfunction, particularly if they are bacterial. Thus, we evaluated whole blood CD64s’s diagnostic performance in neonates with any infection, rather than infection combined with organ dysfunction. Our analysis demonstrated that wbCD64 did not accurately identify all-type bacterial infections in our cohort. While higher wbCD64 levels were associated with bacterial bloodstream infections, the sensitivity of the test for this infection group at less than 60% is unlikely to sufficiently aid decisions to start antimicrobials.

There is a paucity of literature on the measurement of wbCD64, where total CD64 from both neutrophils and monocytes is measured. Monocyte CD64 has low accuracy for infection detection in neonates (36, 43, 68). In prior work in adults, it was hypothesized that a relative abundance of neutrophils to monocytes minimized the impact monocyte CD64 would make on the diagnostic capacity of the test (46). However, it is possible that the contribution of monocyte CD64 masks the diagnostic discrimination capacity of whole blood CD64 in neonates, particularly given typical monocyte counts in this age group have a broader range than adults, and low neutrophil counts are more frequently observed (69). While the ratios of neutrophils to monocytes in our study did not significantly differ between neonates with and without infection, the overall values for the ratios we observed were lower than those described in adult cohorts with infectious and non-infectious morbidities (70–73), and in the limited literature available for neonates (74, 75). A limitation of our study is that we were not able to evaluate the relative contributions of neutrophil and monocyte CD64 to wbCD64 in our study with concurrent flow cytometry. Future work evaluating the contribution of monocyte CD64 to wbCD64 in neonates may have merit, as it is possible that specific measurement of neutrophil CD64 would have improved diagnostic accuracy. Such work could inform further diagnostic development for CD64, particularly given monocyte depletion of whole blood in point of care devices is feasible, having already been established in a CD4 point of care test (Visitect^®^ CD4) (76, 77).

In this study, we included a diverse group of neonates receiving hospital care for the treatment of possible infection. Such a population comprises the heterogeneous presentations a clinician might be expected to encounter in hospital-based neonatal care. However, a neonate’s infection risk profile, inflammatory response to infection, and likelihood of having an alternative diagnosis to infection can each vary depending on factors such as that neonate’s age, gestation, and prior medical history (20, 24). To explore these differences, we examined four subgroups: term and preterm neonates and evaluations for early-onset and late-onset infection. Whole blood CD64 was higher in infants born preterm, and for evaluations for early-onset infections. This observation is not surprising, given well-described hematologic differences (78–80) and differences in the host response to sepsis (20) observed following the first few days after birth. Further, higher neutrophil CD64 levels have previously been described in preterm than term neonates (81), with reduced expression with age in preterm infants (81). While neonates with early-onset infections had some of the highest wbCD64 values, our study only captured three cases in this subgroup, all microbiologically negative. The potential diagnostic performance of wbCD64 for early-onset neonatal infections with microbiologic confirmation thus remains incompletely understood and could be further explored.

This study has several limitations. First, the population was limited to neonates receiving tertiary-level hospital-based care in a high-income country. Our findings may not be directly translatable to other neonatal healthcare contexts, including community-based care for neonates born in situations where hospital care is not feasible (82). Second, due to our approach of using samples already collected for clinical purposes, we necessarily measured wbCD64 in blood refrigerated then frozen rather than fresh samples analyzed at the point of care. Some sample degradation may have occurred in this time, and thus wbCD64 values at point of care may be higher than we observed. Nonetheless, this would be expected to affect all samples similarly and thus the likelihood of introducing bias towards samples from infected versus non-infected episodes due to sample degradation would be very low. However, should wbCD64 be investigated further in the future, we recommend dedicated testing of fresh samples to evaluate wbCD64 values at point of care to allow for measurement without potential sample degradation. Finally, the proportions of infection we observed included a greater proportion of term than preterm infants with infection, in contrast to a known elevated risk of infection associated with prematurity (24). Reasons for this may include a more conservative approach to empiric antimicrobial prescribing for preterm than term neonates, then amplified by our study’s pre-specified aim to recruit similar numbers of preterm and term neonates to ensure both population subgroups were well represented in the cohort. The infection outcome proportions identified in our study thus should not be interpreted as neonatal infection prevalence data.

In conclusion, wbCD64 and NE have a non-linear relationship in neonates. With or without reference to its relationship with NE, wbCD64 is not significantly associated with neonatal infections overall, and does not provide sufficient diagnostic accuracy to aid antimicrobial commencement decisions for neonatal infections. Further characterization of wbCD64 levels over the duration of neonatal infectious illnesses might provide further insights into the potential diagnostic uses for this biomarker, such as antimicrobial cessation decisions.

## Data Availability

The datasets presented in this article are not readily available because data are available on reasonable request, pending approval from the participating institutions. Requests to access the datasets should be directed to naomi.spotswood@burnet.edu.au.
