# Supplementary material for: Evaluation of whole blood CD64 for identifying infection in neonates receiving hospital care
Source: Front Immunol. 2025 Aug 18;16:1629223. doi: 10.3389/fimmu.2025.1629223 (PMC12399554; doi:10.3389/fimmu.2025.1629223)
Supplement: Supplementary file 1 [file Supplementaryfile1.docx]

**Supplement 1**

**ELISA Methods for Neutrophil Elastase and CD64**

Stability testing was performed on 6 samples prior to commencement of this study to evaluate the impact of low volume samples and sample storage conditions for whole blood CD64 and Neutrophil Elastase. No significant differences were noted between higher volume (>250microlitres) compared to low volume (20 microlitre) samples. No significant differences were noted between samples stored at 4^o^C (refrigerated for up to 14 days) compared to -80^0^C (frozen). Frozen samples had slightly reduced signal compared to fresh, however samples frozen immediately or refrigerated then frozen showed no significant difference. Whole blood samples (typically 125 μl) were thawed and mixed with an equal volume of 10% Triton-X100 in de-ionised water for 1 h at 4°C and then stored at 4°C for no more than 48 hours before analysis by ELISA. CD64 was measured using a commercial sandwich ELISA kit (catalogue number SEB578Hu; Cloud-Clone Corp., USA) according to the manufacturer’s recommendations but modified for the whole blood samples. Briefly, microtiter plates pre-coated with anti-human CD64 capture antibodies were incubated at 37°C for 1 hour with 100 μl/well of standard (in a 2-fold dilution series) or lysed whole blood at 1:200 dilution, in duplicate. After removal of the sample, 100 μl of biotinylated anti-human CD64 antibody was added to each well, and the plates further incubated at 37°C for 1 hour. After washings, the plates were incubated with 100 μl/well horseradish peroxidase-avidin (for 30 min at 37°C), further washed and finally 90 μl/well TMB substrate was added for another 20 min of incubation at 37°C. The colour reaction was stopped with 50 μl/well sulphuric acid (H_2_SO4). Absorbance was read at 450/620 nm using a Multiskan microplate reader (ThermoFisher Scientific). Neutrophil Elastase (NE) in lysed whole blood was measured using commercial sandwich ELISA kit (catalogue number DY-9167-05; R&D Systems, USA) according to the manufacturer’s recommendations. Briefly, microtiter plates (Maxisorp, ThermoFischer Scientific, USA) are coated with 100 μl/well mouse anti-human NE capture antibodies and incubated overnight at room temperature. Plates were washed three times and blocked with 300 μl/well PBS 1% BSA for an hour at room temperature before sample addition. Following three washes, the plates were incubated at room temperature for 2 hours with 100 μl/well of standard (in a 2-fold dilution series) or thawed samples (lysed whole blood at 1:10,000 dilution) in duplicate. After removal of the liquid, the plates are washed and 100 μl of biotinylated mouse anti-human NE antibody was added to each well, and the plates further incubated at room temperature for 2 hours. After washings, the plates were incubated with horseradish peroxidase-avidin (for 20 min at room temperature), further washed, developed with TMB substrate and read at 450/620 nm as for the CD64 ELISA.
