# Supplementary material for: Evaluation of whole blood CD64 for identifying infection in neonates receiving hospital care
Source: Front Immunol. 2025 Aug 18;16:1629223. doi: 10.3389/fimmu.2025.1629223 (PMC12399554; doi:10.3389/fimmu.2025.1629223)
Supplement: Supplementary file 2 [file Supplementaryfile2.docx]

Supplement 2

Infection Types and Pathogens Identified

| **Infection Type** | **Evaluations with infection type**  N = 178  n (%) | **Pathogens identified** | **Proportion of all infections**  N = 80  **%** | **Proportion of MCI**  N=55  **%** |
| --- | --- | --- | --- | --- |
| Culture confirmed bloodstream infection, bacterial* | 15 (8.4) | *Staphylococcus aureus*  *Serratia marscescens*  *Streptococcus pneumoniae*  *Escherichia coli*  Coagulase negative staphylococci  *Klebsiella variicola*  *Enterobacter cloacae*  *Streptococcus agalactiae* | 18.8 | 27.3 |
| Microbiologically confirmed meningitis or encephalitis, viral** | 12 (6.7) | Enterovirus  Herpes Simplex Virus  Parechovirus | 15.0 | 21.8 |
| Microbiologically confirmed respiratory tract infection, viral*** | 13 (7.3) | Respiratory Syncytial Virus  Parainfluenza  Human metapneumovirus  Influenza  Rhinovirus | 16.3 | 23.6 |
| Other microbiologically confirmed infection | 15 (8.4) | Coagulase negative staphylococci  *Enterococcus faecalis*  *Escherichia coli*  *Pseudomonas aeruginosa*  *Staphylococcus aureus*  *Serratia marscescens*  *Streptococcus viridans*  Enterovirus  Influenza | 18.8 | 27.3 |
| Necrotising enterocolitis | 3 (1.7) | n/a | 3.8 | n/a |
| Culture negative bloodstream infection | 1 (0.6) | n/a | 1.3 | n/a |
| Culture negative meningitis | 4 (2.2) | n/a | 5.0 | n/a |
| Culture negative respiratory tract infection | 2 (1.1) | n/a | 2.5 | n/a |
| Other infection without microbiologic confirmation | 15 (8.4) | n/a | 18.8 | n/a |
| No Infection | 98 (55.1) | n/a | n/a | n/a |

*Five microbiologically confirmed co-diagnoses which included multi-site infection with the same pathogen (urinary tract, meningitis) and co-infection with a second pathogen (viral, fungal).

**One microbiologically confirmed co-infection at a different site (viral)

***One microbiologically confirmed co-infection at a different site (bacterial)
