# Supplementary material for: Evaluation of whole blood CD64 for identifying infection in neonates receiving hospital care
Source: Front Immunol. 2025 Aug 18;16:1629223. doi: 10.3389/fimmu.2025.1629223 (PMC12399554; doi:10.3389/fimmu.2025.1629223)
Supplement: Supplementary file 3 [file Supplementaryfile3.docx]

Supplement 3

Haematologic Indices and C-Reactive Protein

| Haematologic indices | All evaluations  N=178 Unless otherwise indicated | Any Infection  N= 80  Unless otherwise indicated | No Infection  N=98  Unless otherwise indicated | Unadjusted** | | Adjusted** | |
| --- | --- | --- | --- | --- | --- | --- | --- |
|  |  |  |  | OR  (95% CI) | p-value | OR  (95% CI) | p-value |
| White cell count (x10^9^/L)  median (IQR) | 11.7  (8.9-17.6)  N=177 | 10.9  (8.7-16.5) | 12.6  (9.5-17.6)  N=97 | 0.98  (0.94-1.02) | 0.280 | 0.99  (0.95-1.03) | 0.611 |
| Neutrophil count (x10^9^/L)  median (IQR) | 4.6  (2.9-8.6) | 4.5  (2.4 – 8.0) | 5.3  (3.3-10.1) | 0.98  (0.93-1.04) | 0.466 | 0.98  (0.93-1.04) | 0.587 |
| Monocyte Count (x10^9^/L)  median (IQR) | 1.1  (0.7-1.9) | 1.1  (0.6-1.9) | 1.2  (0.8-1.9) | 0.92  (0.74-1.15) | 0.455 | 0.86  (0.67-1.10) | 0.233 |
| ITR***  median (IQR) | 0.11  (0.05-0.22)  N=113 | 0.13  (0.05-0.33)  N = 39 | 0.10  (0.04-0.19)  N=74 | 1.40****  (1.11-1.78) | **0.005** | 2.59***  (1.45-4.64) | **0.001** |
| CRP >20mg/L  n/N (%) | 27/142  (19.0) | 20/60  (33.3) | 7/82  (8.5) | 5.4  (2.04-14.05) | **0.001** | 2.22  (0.75-6.62) | 0.151 |

*Haematologic indices are from full blood examinations (FBEs) measured from the same sample used to measure wbCD64 and NE. C-reactive protein measurements are included if measured in the 4 hours prior or two hours after the above FBE.

**Logistic regression analyses with the presence of any infection following sepsis evaluation the dependent variable, clustered to account for neonates who contribute datapoints for separate sepsis evaluations, with preterm birth and age >48 hours included in the multivariate (adjusted) model.

***ITR= Immature to Total Ratio of neutrophils. For the regression analysis, these ratios were multiplied by 10, allowing interpretation of the OR for each difference of 0.1 in the ratio.
