# Supplementary material for: Evaluation of whole blood CD64 for identifying infection in neonates receiving hospital care
Source: Front Immunol. 2025 Aug 18;16:1629223. doi: 10.3389/fimmu.2025.1629223 (PMC12399554; doi:10.3389/fimmu.2025.1629223)

Supplement 4

Whole Blood CD64's Correlations with Neutrophil Elastase and Neutrophil Count for Subgroups

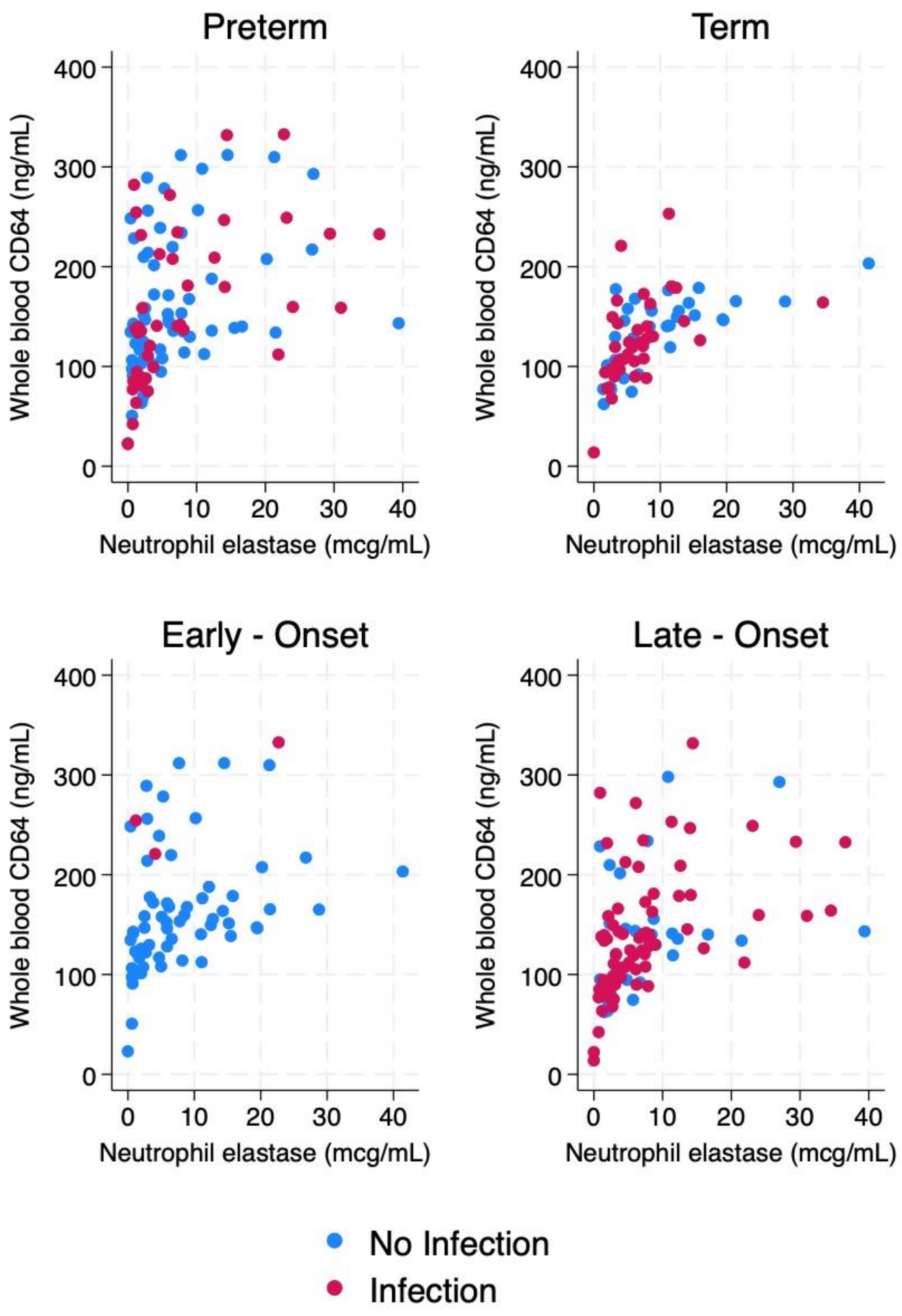

Supplement: Supplementary file 4 [file Supplementaryfile4.pdf]
