# Supplementary figures and images for: Evaluation of whole blood CD64 for identifying infection in neonates receiving hospital care
Source: Front Immunol. 2025 Aug 18;16:1629223. doi: 10.3389/fimmu.2025.1629223 (PMC12399554; doi:10.3389/fimmu.2025.1629223)

## Supplement 5

### Whole Blood CD64's Correlations with Monocyte Count

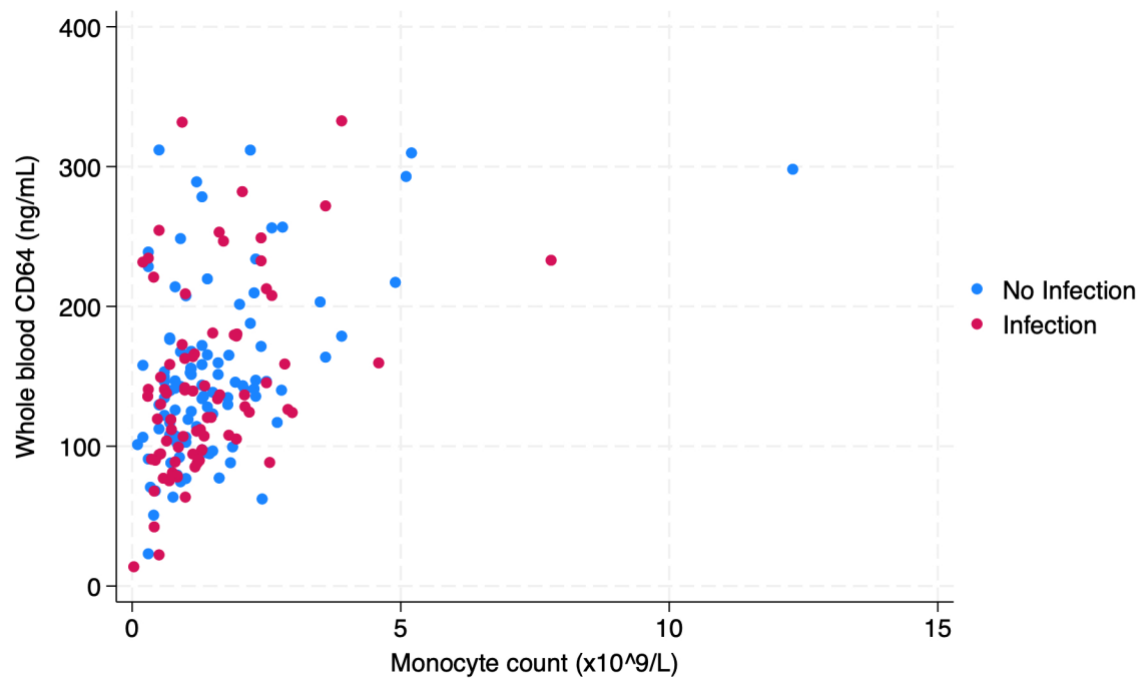

Supplement: Supplementary file 5 [file Supplementaryfile5.pdf]
